# Transcriptomic Profiling of the Maize (*Zea mays* L.) Leaf Response to Abiotic Stresses at the Seedling Stage

**DOI:** 10.3389/fpls.2017.00290

**Published:** 2017-03-01

**Authors:** Pengcheng Li, Wei Cao, Huimin Fang, Shuhui Xu, Shuangyi Yin, Yingying Zhang, Dezhou Lin, Jianan Wang, Yufei Chen, Chenwu Xu, Zefeng Yang

**Affiliations:** Jiangsu Provincial Key Laboratory of Crop Genetics and Physiology/Co-Innovation Center for Modern Production Technology of Grain Crops, Key Laboratory of Plant Functional Genomics of Ministry of Education, Yangzhou UniversityYangzhou, China

**Keywords:** *Zea mays* L., abiotic stress, RNA-Seq, differentially expressed genes (DEGs), expression pattern

## Abstract

Abiotic stresses, including drought, salinity, heat, and cold, negatively affect maize (*Zea mays* L.) development and productivity. To elucidate the molecular mechanisms of resistance to abiotic stresses in maize, RNA-seq was used for global transcriptome profiling of B73 seedling leaves exposed to drought, salinity, heat, and cold stress. A total of 5,330 differentially expressed genes (DEGs) were detected in differential comparisons between the control and each stressed sample, with 1,661, 2,019, 2,346, and 1,841 DEGs being identified in comparisons of the control with salinity, drought, heat, and cold stress, respectively. Functional annotations of DEGs suggested that the stress response was mediated by pathways involving hormone metabolism and signaling, transcription factors (TFs), very-long-chain fatty acid biosynthesis and lipid signaling, among others. Of the obtained DEGs (5,330), 167 genes are common to these four abiotic stresses, including 10 up-regulated TFs (five ERFs, two NACs, one ARF, one MYB, and one HD-ZIP) and two down-regulated TFs (one b-ZIP and one MYB-related), which suggested that common mechanisms may be initiated in response to different abiotic stresses in maize. This study contributes to a better understanding of the molecular mechanisms of maize leaf responses to abiotic stresses and could be useful for developing maize cultivars resistant to abiotic stresses.

## Introduction

Maize (*Zea mays* L.), a widely grown staple food, feed and industrial crop, plays a critical role in supporting the growing world population. The production process of maize is highly dependent on suitable environmental factors (Gong et al., 2014). However, reduction in the availability and quality of arable land and water resources as well as frequent extreme weather can cause many different types of abiotic stresses, such as salinity, drought, and extreme temperatures (heat, cold, and freezing) (Krasensky and Jonak, 2012). These major abiotic environmental stressors seriously affect crop development and constrain agronomical yield worldwide. Abiotic stresses may be responsible for a yield reduction of over 50% in major crop plants globally (Mahajan and Tuteja, 2005; Funk and Brown, 2009; Rodziewicz et al., 2014; de Zelicourt et al., 2016). In China, 60% of the maize plants are located in arid areas, and a 20–30% yield loss per year occurs in maize due to drought (Gong et al., 2014). The seedling stage of maize is especially sensitive to abiotic stresses; seedling damage can lead to stunted development and death (Peleg and Blumwald, 2011), resulting in reduced production or even rejection and incurring significant economic costs. Thus, seedling damage from abiotic stress is a subject of great concern in maize production.

Abiotic stresses can induce physiological, molecular and biochemical changes that disturb various cellular and whole-plant processes, which in turn negatively influence the development and yield of crops. Cell membranes may become disorganized, osmotic stress could be altered, proteins may lose activity or be denatured, and high levels of reactive oxygen species (ROS) could result in oxidative damage (de Zelicourt et al., 2016). These cellular changes can result in damaged cell membrane integrity, restrained photosynthesis, and dysfunctional metabolism, all of which subsequently disturb growth and development, reduce fertility, and induce premature senescence and even death of crops (Krasensky and Jonak, 2012). To counter these negative impacts of abiotic stresses, crops have evolved sophisticated resistance mechanisms in response to various stress factors, such as stress avoidance and stress tolerance. Stress avoidance is a protective mechanism that can help crops prevent or delay the negative impact of abiotic stresses, while stress tolerance is the acclimation of plants to stressful conditions (Krasensky and Jonak, 2012). Crop responses to various abiotic stresses occur at all levels of organization, including cellular responses, metabolic changes, and transcriptional regulation of gene expression. At the cellular level, crops can adjust membrane systems and modify the cell wall architecture. Several compatible solutes (e.g., proline, raffinose) can be produced to help stabilize proteins and cellular structures (Valliyodan and Nguyen, 2006; Munns and Tester, 2008). One of the fastest metabolic responses of crops is the biosynthesis of abscisic acid (ABA), which can regulate stomatal closure to reduce water loss to maintain cellular growth (Peleg and Blumwald, 2011). All of these responses are controlled at the molecular level by regulating the expression of genes involved in the synthesis of osmoprotectants and transporters and of genes encoding regulatory proteins such as protein kinases, phosphatases, and transcription factors (TFs; Sakuma et al., 2006; Krasensky and Jonak, 2012). Significant progress has been made in understanding the molecular mechanisms of plant responses to abiotic stress factors. ABA is called a “stress hormone,” the discovery of ABA synthesis, perception, signaling, and transportation is a breakthroughs to understand the essential for the ability of plants to adapt to abiotic stresses (Peleg and Blumwald, 2011; Qin et al., 2011). The functions of TFs in stress tolerance have received much attention, many TFs belonging to AP2/EREBP, MYB, WRKY, NAC, bZIP families have been found to be involved in various abiotic stresses and some TF genes have also been engineered to improve stress tolerance in model and crop plants (Qin et al., 2011; Wang H.Y. et al., 2016). In addition, hormone cross-talk and lipid signaling also play a vital role in response to abiotic stresses (Peleg and Blumwald, 2011; Qin et al., 2011; Hou et al., 2016). Improvement of resistance to abiotic stresses is considered to be the most cost-effective management approach to prevent or reduce the hazards of abiotic stresses in maize (Mahajan and Tuteja, 2005). However, the molecular mechanisms of resistance to various abiotic stresses in maize are not well understood.

Abiotic stresses, especially salinity, drought, heat, and cold stress, are becoming the major threat to yield in the primary maize production regions. Utilization of maize cultivars with desirable resistance to salinity, drought, heat, or cold stress is the most cost-effective approach for preventing stress damage. Resistance to salinity, drought, heat, and cold stress in maize is a very complex abiotic stress-responsive mechanism. Investigations of the molecular mechanisms of maize response to salinity, drought, heat, or cold stress are needed to facilitate the development of elite resistant maize varieties and more effective management strategies. RNA-seq is a powerful technology for whole genome gene expression profile analysis and is especially useful for studying complex gene regulatory networks (McGettigan, 2013). To gain a comprehensive understanding of the molecular mechanisms involved in the response to salinity, drought, heat, or cold stress in the seedling stage of maize, RNA-seq was used to obtain the transcriptomic profiles of B73 seedling leaves in response to salinity, drought, heat, or cold stress at the whole-genome level. Differentially expressed genes (DEGs) were identified by comparisons between the control and the abiotic stress samples, and these DEGs were compared between the different stress samples to detect the unique and common genes and pathways responding to different abiotic stresses in maize. This study advances the understanding of the molecular responses to abiotic stresses in maize, which could lead to improved strategies for the development of new resistant maize cultivars.

## Materials and Methods

### Plant Material and Treatments

B73 maize seedling plants were grown in plant growth chambers with controlled conditions: 28/22°C during a 14/10 h light/dark cycle, light density of 250–300 mmol m^-2^ s^-1^. Each black plastic pot (10.0 cm × 10.0 cm) was filled with soil and watered every 2 days. When the third leaves were fully expanded, the plants were subjected to salinity, drought, heat or cold. For high salt stress, plants were watered with 200 mM NaCl for 2 h prior to tissue collection. For drought treatment, the 6-day-old maize seedlings were grown without watering until their third leaves were fully expanded. For heat and cold stress treatments, seedlings were incubated at 42°C and 4°C for 2 h, respectively. After treatments, the third leaves were collected and immediately stored in liquid nitrogen for further analysis. Two independent experimental replicates were performed.

### RNA Isolation and cDNA Library Construction

The leaves sampled from control and stress treatments were collected for RNA isolation and cDNA library construction. Two replicates were prepared for each sample, resulting in 10 libraries that were used for transcriptome sequencing using the Illumina HiSeq X Ten system.

Total RNA of maize seedling leaves was isolated using an RNeasy^®^ Plant Mini kit (Qiagen, Shanghai, China), according to the manufacturer’s protocol. All RNA samples were treated with RNase-free DNase I. A NanoDrop^®^ 2000 spectrophotometer (Thermo Scientific, Wilmington, DE, USA), a Qubit^®^ Fluorometer 2.0 (Life Technologies, Carlsbad, CA, USA) and an Agilent 2100 bioanalyzer (Agilent Technologies, Santa Clara, CA, USA) were used to test the concentration and integrity of RNA samples, and confirm that all RNA samples had an integrity value >6.5.

### Mapping of Sequencing Reads and Quantification of Gene Expression

The clean data were obtained by removing adapters, low-quality reads, and reads containing poly-N from the raw data. The Q20 and Q30 values, GC content, and sequence duplication levels were calculated for the clean data. The clean data were used for further analysis. The sequencing data were deposited in the NCBI Short Read Archive database with the accession number SRP080208.

High quality reads were aligned to the B73 reference sequence (AGPv3 release 31^1^) using TopHat (v2.0.9). HTSeq (v0.5.3) was used to count the read numbers mapped to each gene (Robinson and Oshlack, 2010; Anders et al., 2015). The FPKM (Fragments Per Kilobase of exon model per Million mapped reads) of each gene was calculated based on the length of the gene and read count mapped to it (Mortazavi et al., 2008).

### Expression Analysis and Enrichment Analysis of Differentially Expressed Genes

The DEGs were detected with the Bioconductor package ‘edgeR’ in R between control and stress samples. The resulting *p*-values were adjusted using Benjamini and Hochberg’s approach to control the false discovery rate (Benjamini and Hochberg, 1995). Genes with an adjusted *p*-value (*q*-value) ≤ 0.05 and an absolute value of log_2_ fold changes (FC) ≥ 1 were considered as differentially expressed.

Gene Ontology (GO) and Kyoto Encyclopedia of Genes and Genomes (KEGG) analyses were performed to identify which DEGs were significantly enriched in GO terms or metabolic pathways. GO enrichment analysis of the DEGs was conducted using GOseq R packages based on the Wallenius non-central hyper-geometric distribution (Young et al., 2010), which can adjust for gene length bias in DEGs. GO terms with corrected *p*-value (*q*-value) ≤ 0.05 were considered significantly enriched among the DEGs. KOBAS (v2.0.12) software was used to enrich the DEGs in the KEGG pathways (Mao et al., 2005). A corrected *p*-value (*q*-value) ≤ 0.05 was the threshold for significantly enriched KEGG pathways. The web-based system Plant MetGenMAP was used to assign DEGs to metabolic pathways (Joung et al., 2009). The Plant Transcription Factor Database v3.0 was used to assign DEGs to different TF families^2^.

### qRT-PCR Analysis

To validate the repeatability of gene expression obtained by RNA-seq, eight DEGs were randomly selected for validation by qRT-PCR. Independent RNA with two replicates of the maize seedling leaves from control and four abiotic stresses was prepared for qRT-PCR analysis. RNA extraction and quality control were performed as described above. Gene specific primers (Supplementary Table S1) were designed according to the sequences of the eight genes using QuantPrime^3^. The relative expression levels of the genes were calculated using the 2^-ΔΔCt^ method (Pfaffl, 2001; Schmittgen and Livak, 2008), which represents the C_T_ (cycle threshold) difference between the reference *Actin* gene and the target gene product.

## Results

### RNA-Seq and Transcriptome Profiles of Maize Leaves in Response to Abiotic Stresses

**FIGURE 1:**
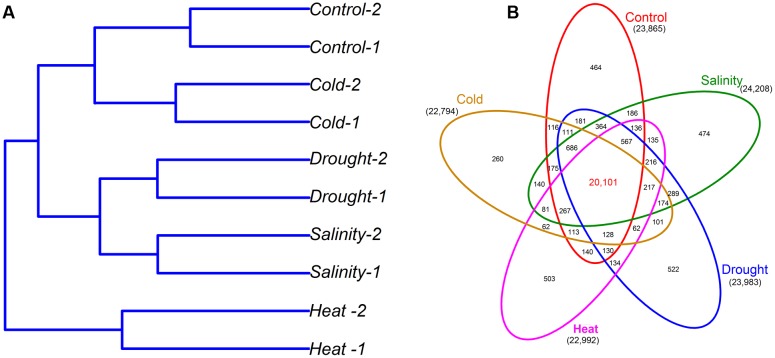
**Hierarchical clustering of the 10 RNA-seq samples based on Euclidian distance (A)**, and overlap of active genes under different stress conditions **(B)**.

Seedling plants of the maize inbred line B73 were either subjected to salinity, drought, heat, and cold stress conditions or grown in normal conditions (control). The total RNA of leaves from these seedling plants was sequenced using an Illumina system. We performed transcriptomic analysis of the five samples (Control, Salinity, Drought, Heat, and Cold), with two biological replicates for each condition, to profile the maize response to abiotic stresses. The RNA-seq analysis yielded 46.0–68.0 million raw reads per biological replicate with an average read length of 150 bp (**Table 1**). After filtering out adapter and low-quality sequences, approximately 74.4 Gb of clean bases were obtained in the 10 transcriptome libraries. Of the clean reads, 77.54–82.97% were uniquely mapped to the maize reference genome sequence. The normalized FPKM was used to quantify the gene expression level (Trapnell et al., 2010). Pearson correlation analysis (Supplementary Table S2) and hierarchical cluster analysis (**Figure 1A**) revealed that the gene expression data of the five samples were highly reproducible between the two biological replicates of each sample, and the transcriptomes of maize seedling leaves under drought and salinity stress clustered in close proximity to each other, while the maize transcriptome under heat stress formed a separated cluster.

**Table 1:** Summary of the sequence data from Illumina sequencing.

Sample	Replicate	Raw reads	Clean reads	Mapped reads	Mapped unique reads	Mapping ratio
Control	1	54,477,760	49,675,389	44,635,503	43,244,605	89.85%
Control	2	68,407,916	62,928,805	56,727,053	54,894,173	90.14%
Salinity	1	58,631,264	54,058,240	50,067,776	48,648,028	92.62%
Salinity	2	56,479,100	51,697,101	46,452,250	45,107,142	89.85%
Drought	1	50,139,498	45,461,544	40,658,089	39,532,523	89.43%
Drought	2	48,237,130	43,045,649	38,406,511	37,403,429	89.22%
Heat	1	52,812,100	47,787,827	43,793,437	42,479,949	91.64%
Heat	2	53,198,796	48,311,290	43,055,090	41,908,330	89.12%
Cold	1	55,216,014	50,007,363	44,881,791	43,377,179	89.75%
Cold	2	46,954,284	42,754,619	37,962,983	36,756,281	88.79%


*Mapping ratio = Mapped reads/All reads*.

A gene was considered to be active when more than five reads were uniquely mapped on the maize reference genome sequence under at least one condition. In total, 27,235 of 39,625 genes were active (**Figure 1B**). Among the active genes (27,235), 24,208 were expressed during salinity stress, 23,983 during drought stress, 22,992 during heat stress, and 22,794 during cold stress, while 23,865 were expressed in the control condition. A total of 20,101 genes were expressed in all five samples, while 1,111 genes were specifically expressed in only one sample. The Control, Salinity, Drought, Heat, and Cold samples contained 464, 474, 522, 503, and 260 specifically expressed genes, respectively. Among the drought stress-specific genes, six GO terms (GO:0009607, response to biotic stimulus; GO:0010876, lipid localization; GO:0006869, lipid transport; GO:0030243, cellulose metabolic process; GO:0030244, cellulose biosynthetic process; GO:0005976, polysaccharide metabolic process) were significantly enriched, and the salinity stress-specific genes were significantly enriched in four GO terms (GO:0016684; oxidoreductase activity; GO:0004601, peroxidase activity; GO:0016209, antioxidant activity; GO:0030528, transcription regulator activity). However, the specifically expressed genes of heat and cold stresses did not show enrichment in any GO term.

### Identification and Analysis of Stress-Specific Differentially Expressed Genes (DEGs)

**FIGURE 2:**
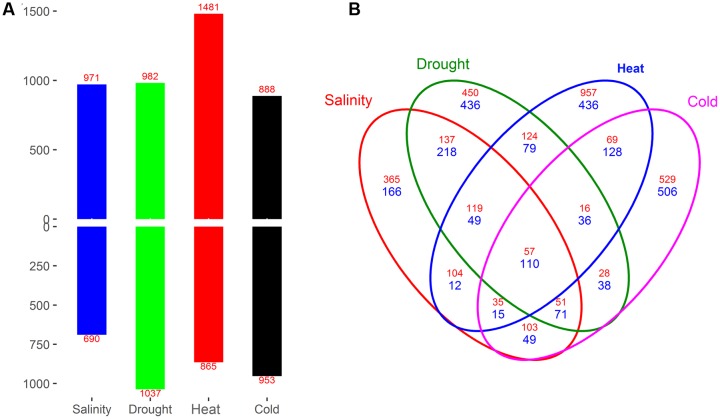
**Numbers of differentially expressed genes (DEGs) in the four pairwise comparisons of the control and stress treatments (A)**, and overlap between DEGs **(B)**. DEGs from Salinity vs. Control, Drought vs. Control, Heat vs. Control, and Cold vs. Control comparisons are indicated by Salinity, Drought, Heat, and Cold, respectively. The number of up-regulated and down-regulated genes is indicated by red and blue font, respectively.

Each stress sample was compared with the control to identify DEGs (*q*-value ≤ 0.05 and |log_2_ FC|≥ 1), and 5,330 DEGs were obtained from these four comparisons (Salinity vs. Control, Drought vs. Control, Heat vs. Control, and Cold vs. Control). From these pairwise comparisons with the control sample, we identified 1,661 (971 up- and 690 down-regulated) DEGs in the salinity stress sample, 2,019 (982 up- and 1,037 down-regulated) in the drought stress sample, 2,346 (1481 up- and 865 down-regulated) in the heat stress sample and 1,841 (888 up- and 953 down-regulated) in the cold stress sample. Under salinity and heat stress, the number of up-regulated DEGs was much higher than the number of down-regulated genes (**Figure 2A**).

By comparing the four stress responses at the gene level, 167 DEGs were identified that were common to salinity, drought, heat, and cold stress, including 57 up- and 110 down-regulated genes (**Figure 2B**). We also identified hundreds of genes specific for salinity (365 up- and 166 down-regulated), drought (450 up- and 436 down-regulated), heat (957 up- and 436 down-regulated) and cold stress (529 up- and 506 down-regulated).

### Functional Classification of DEGs

A GO analysis was performed to determine the function of the identified DEGs. The 5,330 DEGs were enriched in 51 GO terms in the molecular function and biological process categories (Supplementary Figure S1). Four GO terms included more than 500 DEGs. Catalytic activity (GO:0003824) and transferase activity (GO:0016740), with 1,661 and 588 DEGs, respectively, were the two most dominant terms in the molecular function category, while metabolic process (GO:0008152) and biological regulation (GO:0065007), with 1,916 and 653 DEGs, respectively, were the first two major terms in the biological process category.

**FIGURE 3:**
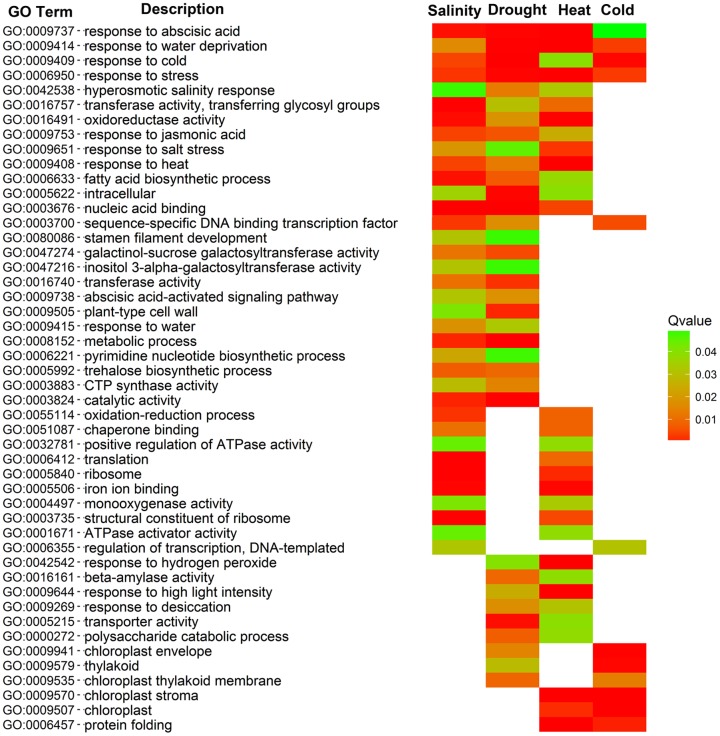
**Cross-comparison of enriched GO terms among DEGs in response to Salinity, Drought, Heat, and Cold.** Different colors in the right columns represent the different significance levels (*q*-values) of the overrepresentation. DEGs from Salinity vs. Control, Drought vs. Control, Heat vs. Control, and Cold vs. Control comparisons are indicated by Salinity, Drought, Heat, and Cold, respectively.

Among the DEGs identified between the stress and control samples, 70, 84, 46, and 19 GO terms were enriched in the comparison of salinity, drought, heat, and cold stress versus control, respectively. To determine the transcriptomic changes that occur in response to various abiotic stresses, the enriched GO terms under different stress conditions were compared, and all commonly enriched GOs are summarized in **Figure 3**. Four GO terms (GO:0009737, response to ABA; GO:0009414, response to water deprivation; GO:0009409, response to cold and GO:0006950, response to stress) were enriched in all datasets, and all four were related to stimulus responses. In addition, 10 and 34 GO terms were commonly enriched in maize leaves under three and two types of abiotic stresses, respectively. There were 105 GO terms uniquely enriched in one comparison (Salinity vs. Control, Drought vs. Control, Heat vs. Control, or Cold vs. Control).

To further investigate the biological functions of these DEGs, pathway-based analysis was conducted using KEGG. We identified 11 pathways that were significantly enriched in comparisons of stress samples versus the control (Salinity vs. Control, Drought vs. Control, Heat vs. Control, or Cold vs. Control), including two pathways enriched in two comparisons and nine stress-specific pathways (Supplementary Figure S2). It is worth noting that the “Protein processing in endoplasmic reticulum” pathway was enriched in the Drought vs. Control comparison, and several genes in the “Protein processing in endoplasmic reticulum” pathway were differentially expressed in maize seedling leaves under salinity, heat, and/or cold stresses. Moreover, the “Carbon metabolism,” “starch and sucrose metabolism,” and “carbon fixation” pathways were significantly enriched in maize seedling leaves in response to heat stress (**Supplementary Figure S2**).

### Dynamic Expression of Transcription Factors in Response to Abiotic Stresses

**FIGURE 4:**
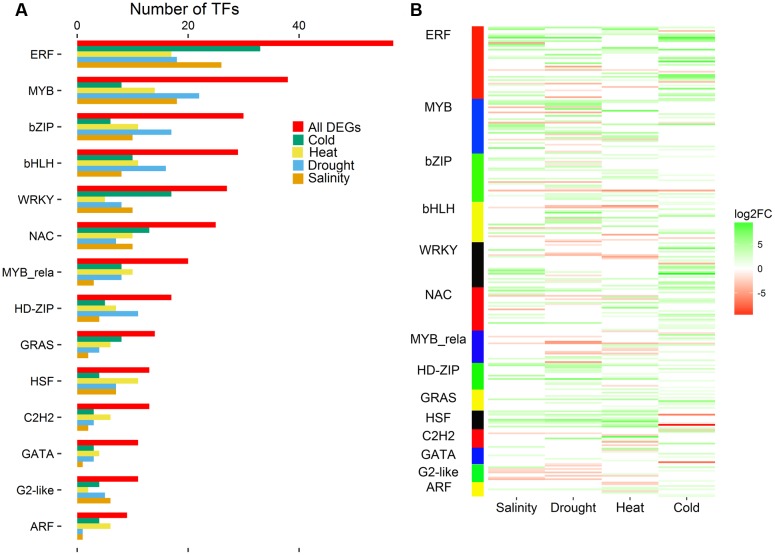
**Distribution of DEGs into 14 major transcription factor families that include more than nine DEGs (A)**. Heatmap of differentially expressed transcription factors in the four pairwise comparisons of control and stress treatments **(B)**. Expression values of genes are presented as FPKM-normalized log_2_-transformed counts. Green and red colors indicate up- and down-regulated transcripts, respectively. DEGs from Salinity vs. Control, Drought vs. Control, Heat vs. Control, and Cold vs. Control comparisons are indicated by Salinity, Drought, Heat, and Cold, respectively.

Next, the DEGs encoding TFs were analyzed. A total of 403 DEGs encoding TFs were identified in maize seedling leaves in response to salinity, drought, heat, and cold abiotic stresses, and these TFs belonged to 43 TF families. Most of the identified DEGs encoded members of the ERF, MYB, bZIP, bHLH, WRKY, NAC and MYB-related TF families (**Figure 4A**), and 14 TF families included more than 9 differentially expressed TFs. The ERF family, with 58 DEGs, was the largest TF family responding to abiotic stresses, including 26, 18, 17, and 33 DEGs in maize seedling leaves under salinity, drought, heat, and cold stress, respectively. A total of 38 DEGs belonging to the MYB family were identified, including 18, 22, 14, and 8 DEGs in maize leaves under salinity, drought, heat, and cold stress conditions, respectively. Most of the differentially expressed *ERFs* and *MYBs* were up-regulated; conversely, *G2*-like and *ARF*s were typically down-regulated (**Figure 4B**). The TFs had different expression patterns in maize seedling leaf responses to salinity, drought, heat and cold stresses, which suggested that maize possesses a wide variety of abiotic stress resistance mechanisms.

### Expression of Hormone Biosynthesis and Signal Transduction Genes in Response to Different Abiotic Stresses

**FIGURE 5:**
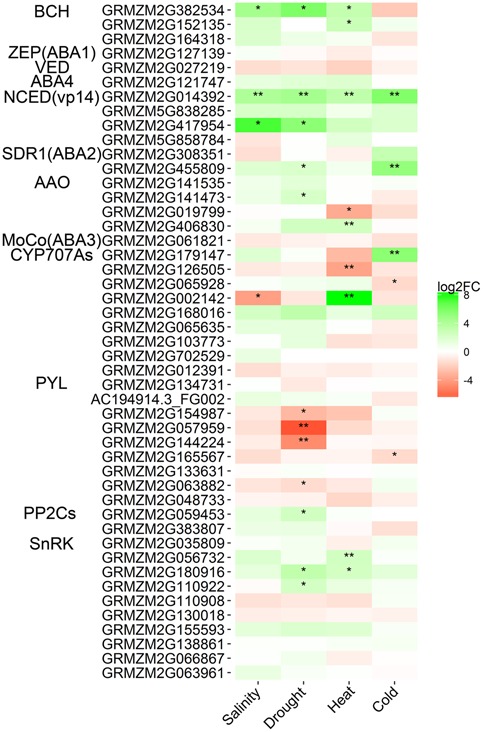
**Heatmap of DEGs involved in the ABA biosynthesis and signal transduction pathways in the four pairwise comparisons of control and stress treatments.** Expression values of genes are presented as FPKM-normalized log_2_-transformed counts. Green and red colors indicate up- and down-regulated transcripts, respectively. DEGs from Salinity vs. Control, Drought vs. Control, Heat vs. Control, and Cold vs. Control comparisons are indicated by Salinity, Drought, Heat, and Cold, respectively. ^∗^*Q* < 0.05; ^∗∗^*Q* < 0.001.

**FIGURE 6:**
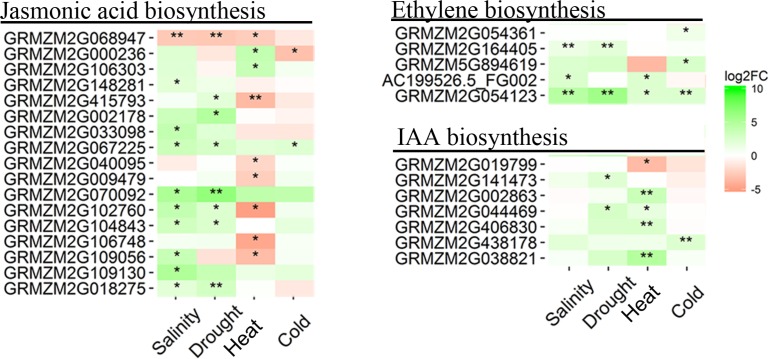
**Heatmap of DEGs involved in hormone (jasmonic acid, ethylene, and IAA) biosynthesis, photosynthesis, and carbon metabolism pathways in the four pairwise comparisons of control and stress treatments.** Expression values of genes are presented as FPKM-normalized log_2_-transformed counts. Green and red colors indicate up- and down-regulated transcripts, respectively. DEGs from Salinity vs. Control, Drought vs. Control, Heat vs. Control, and Cold vs. Control comparisons are indicated by Salinity, Drought, Heat, and Cold, respectively. ^∗^*Q* < 0.05; ^∗∗^*Q* < 0.001.

Approximately, 50 DEGs involved in hormone biosynthesis and signal transduction pathways, such as the ABA, jasmonic acid (JA), ethylene (ET), and auxin (IAA) pathways, were identified. Moreover, 21 DEGs encoding ABA biosynthesis and catabolism enzymes and ABA receptors were obtained in comparisons between stress samples and the control (**Figure 5**). As expected, the expression levels of most ABA biosynthesis enzyme genes were up-regulated in maize seedling leaves under salinity, drought, heat, and cold stresses, including *BCH* (β-carotene hydroxylase) and *NECD* (9-*cis*-epoxycarotenoid dioxygenase). Two up-regulated *BCH*s (GRMZM2G382534 and GRMZM2G152135) were identified, with GRMZM2G382534 showing up-regulation under salinity, drought and heat stress conditions. The expression levels of *ABA1*, *VED* (violaxanthin de-epoxidase) and *ABA4* were not significantly affected by salinity, drought, heat, or cold stress. Two up-regulated *NECD*s were identified, including *vp14* (GRMZM2G014392), the first cloned *NECD* gene in maize, which was significantly induced 10.7- to 60.8-fold in all four stress samples. The transcriptomic levels of *AAO*s slightly increased in maize seedling leaves in response to different treatments. *CYP707A*s, encoding ABA catabolism enzymes, showed different expression patterns in the four treatment samples. However, the expression levels of three DEGs encoding ABA effectors were determined in response to salinity, drought, heat, and cold stresses. Four *PYR1/PYL*s displayed obviously decreased expression levels in drought stress, while only one *PYR1/PYL* was significantly down-regulated in cold stress, and none of the *PYR1/PYL*s were affected by salinity or heat treatment. One *PP2C* and three *SnRK*s of maize seedling leaves were up-regulated under drought or heat stress. We also detected 17, 5, and 7 DEGs involved in the JA, ET, and IAA biosynthesis pathways, respectively. Most of these phytohormone biosynthesis-related genes were up-regulated in maize seedling leaves in response to abiotic stresses, but their expression levels clearly varied by treatment condition (**Figure 6** and **Supplementary Dataset S2**). These results indicated that phytohormone biosynthesis was reprogrammed under different abiotic stress conditions.

### Very-Long-Chain Fatty Acid and Lipid Signaling in Response to Abiotic Stresses

**FIGURE 7:**
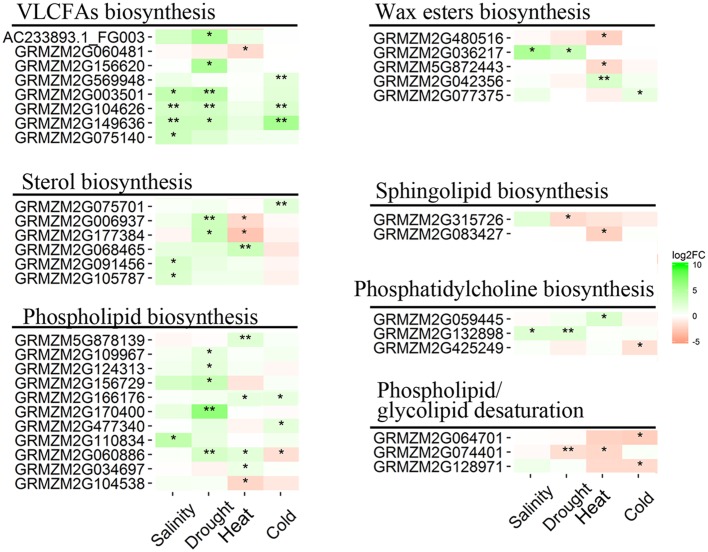
**Heatmap of DEGs involved in very-long-chain fatty acid (VLCFA) and lipid signaling in the four pairwise comparisons of control and stress treatments.** Expression values of genes are presented as FPKM-normalized log_2_-transformed counts. Green and red colors indicate up- and down-regulated transcripts, respectively. DEGs from Salinity vs. Control, Drought vs. Control, Heat vs. Control, and Cold vs. Control comparisons are indicated by Salinity, Drought, Heat, and Cold, respectively. ^∗^*Q* < 0.05; ^∗∗^*Q* < 0.001.

Cuticular waxes, consisting mostly of very-long-chain fatty acids (VLCFAs) and their derivatives, play crucial roles in protecting plants against abiotic stresses (Wang et al., 2015; Wang M.L. et al., 2016). In this study, 13 DEGs involved in VLCFA and wax ester biosynthesis pathways were identified (**Figure 7**), including 10 genes that were up-regulated and 3 that were down-regulated in response to salinity, drought, heat and cold stresses. The expression levels of these 10 up-regulated DEGs increased 3.0- to 45.8-fold in stress samples, and the expression levels of the three down-regulated DEGs decreased 3.7- to 5.2-fold in stress samples (**Supplementary Dataset S2**). Among the DEGs, eight genes encoding ketoacyl-CoA synthase (KCS) were identified, which were mainly up-regulated by abiotic stress. Two transcripts for fatty acyl-coA reductase (FAR) involved in the wax ester biosynthesis pathway were found in DEGs. Of these two FAR transcripts, GRMZM2G036217 was up-regulated by salinity and drought stress, while GRMZM2G480516 was down-regulated by cold stress. Phosphatidic acid, phosphoinositides, sphingolipids and other lipids are involved in the resistance to abiotic and biotic stresses in plants (Hou et al., 2016). In this study, 25 DEGs involved in sterol, sphingolipid, phospholipid, and phosphatidylcholine biosynthesis and in phospholipid/glycolipid desaturation pathways were identified in maize seedling leaves in response to salinity, drought, heat and cold stresses (**Figure 7** and **Supplementary Dataset S2**).

### Validation of RNA-Seq Analysis by Quantitative Real-Time PCR (qRT-PCR)

**FIGURE 8:**
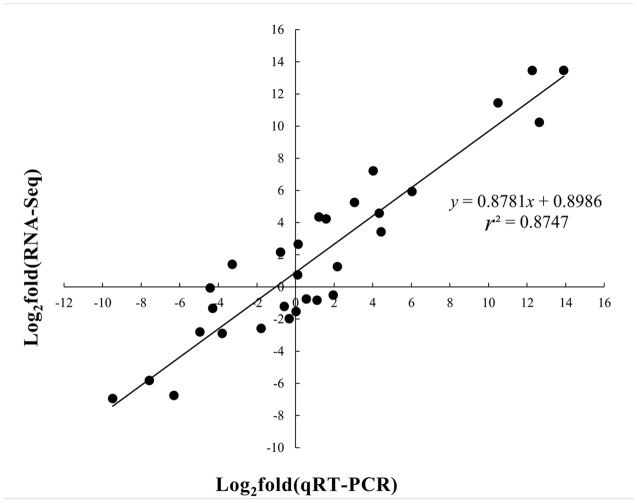
**Quantitative real-time PCR validations of DEGs characterized by RNA-seq**.

To validate the reliability of the gene expression data obtained by the RNA-seq analysis in maize seedling leaves, eight DEGs were randomly selected from the control and the stress samples for qRT-PCR analysis (Supplementary Table S1). The ratio of expression levels found between stress samples and the control using qRT-PCR was compared to the ratio of expression as measured by RNA-Seq. A significant correlation (*r*^2^ = 0.8747, *n* = 30, **Figure 8**) was observed between the RNA-Seq and qRT-PCR data, which confirmed the authenticity of the DEGs in this study. Thus, these comparisons of data from qRT-PCR and RNA-seq analyses of B73 seedling leaves fully validated the findings from our transcriptome study.

## Discussion

Abiotic stresses (such as drought, salinity, heat, and cold) strongly affect crop development and yield. Worldwide, abiotic stresses cause major crop production decreases of over 50%. A better understanding of the molecular mechanisms involved in the maize response to abiotic stresses would facilitate the development of stress resistant cultivars. RNA-seq is a useful approach for the identification of DEGs and regulatory mechanisms at the transcriptome level, which could provide insights into the molecular basis of the maize response to abiotic stresses (Kakumanu et al., 2012; Opitz et al., 2014; Frey et al., 2015; Zhang et al., 2015).

In this study, we selected the period of third leaves, because that it have been suggested that the seedling stage of maize is especially sensitive to abiotic stresses (Peleg and Blumwald, 2011). Three-leaf stage represents the full immature-to-mature gradient of variation in morphology, anatomy and gene expression as the leaf is undergoing functional differentiation (Li et al., 2010). An average of 23,568 active genes were identified by transcriptomic analysis in maize seedling leaves under different conditions. Interestingly, the number of active genes in each treatment sample was comparable (**Figure 1**). This result was similar to two previous studies (Li et al., 2010; Opitz et al., 2016), which examined 9-day-old B73 leaves and 4-day-old maize seedling roots (under water deficit conditions), respectively. However, the number of active genes obtained in our study was slightly higher than that obtained by Frey from maize leaves under heat stresses (Frey et al., 2015). In our study, 20,101 genes were expressed in all four treatments, and 260–522 genes were specifically expressed in one treatment. In total, 5,330 DEGs were identified between the control and stress samples by pairwise comparisons. The largest effect of abiotic stress on gene regulation was detected under heat stress, with 1,481 up- and 865 down-regulated DEGs identified. The number of up-regulated DEGs was much higher than the down-regulated genes under salinity and heat stress, which is consistent with the results of transcriptomic profiling of European maize inbred lines (Frey et al., 2015) and *Medicago falcata* (Miao et al., 2015). Functional annotation of DEGs found that a number of biological pathways, including hormone metabolism and signaling, transcriptional regulation and lipid signaling, participated in the response to abiotic stress in seedling maize (**Figures 3**–**7**).

### DEGs Involved in ABA Biosynthesis and Signaling

Plant hormones play critical roles in regulating the growth and development of crops under abiotic stresses (Peleg and Blumwald, 2011). One of the most studied phytohormones is ABA, which has been described as a stress hormone, and crops have been shown to adjust ABA levels in response to adverse environmental conditions (Tuteja, 2007). ABA biosynthesis and catabolism are regulated by BCH, ABA1, NCED, ABA2, AAO, and CYP707A (Nambara and Marion-Poll, 2005; Peleg and Blumwald, 2011; Chan, 2012; Shan et al., 2013; Shi et al., 2014; Song et al., 2014; Miao et al., 2015; Shankar et al., 2016). Zeaxanthin is an important intermediate in the biosynthetic reaction of β-carotene to form ABA; β-carotene hydroxylases encoded by *BCH* can catalyze the biosynthesis of zeaxanthin (Finkelstein, 2013); xanthophyll cleavage by NCED is the first committed, rate-limiting step in ABA biosynthesis; and CYP707As encode enzymes that can catalyze ABA catabolism. The expression of these genes involved in ABA biosynthesis and catabolism could be activated or decreased by abiotic stresses (Tuteja, 2007; Song et al., 2014; Miao et al., 2015). In our study, the expression levels of *BCH*, *NECD*, *ABA2*, and *AAO*, the key genes involved in ABA biosynthesis, were significantly up-regulated in seedling maize leaves in response to salinity, drought, heat, and cold stresses. Increased expression levels of the *NCED* genes have been shown in both roots and leaves under abiotic stress conditions, especially in maize (Tan et al., 1997, 2003; Iuchi et al., 2001; Xiong et al., 2001). *Vp14* was the first cloned *NCED* gene in maize (Tan et al., 1997), and its expression is significantly up-regulated by most abiotic stresses (Shan et al., 2013; Song et al., 2014; Miao et al., 2015). ABA levels in crops are the result of a delicate balance between ABA biosynthesis and catabolism (Nambara and Marion-Poll, 2005). CYP707A is an important hydroxylase involved in ABA catabolism (Finkelstein, 2013), and the *CYP707A* genes showed different expression patterns in response to salinity, drought, heat and cold stresses in this study (**Figure 5**). Different spatial and temporal expression patterns of CYP707A have suggested that CYP707As could play different physiological roles in plant development (Nambara and Marion-Poll, 2005). Our observation that genes involved in ABA biosynthesis were up-regulated in response to stress indicates that the endogenous ABA level in seedling maize increases to adapt to various abiotic stresses.

Expression of components of the ABA signaling pathway, such as PYR/PYL, PP2C, and SnRK, is related to various abiotic stresses in crops (Nambara and Marion-Poll, 2005; Finkelstein, 2013; Fan et al., 2016). The expression levels of *PP2C*s and *SnRK*s were mainly up-regulated, while *PYR/PYL*s showed down-regulation, in maize (Fan et al., 2016) and *Arabidopsis* (Chan, 2012). Five *PYR/PYL* DEGs were identified in this study, including four in response to drought stress in seedling maize leaves and one in response to cold stress (**Figure 5**). In addition, only one *PP2C* was identified in drought stress, and three *SnRK*s were differentially expressed in drought and heat stress conditions. Consistent with previous reports, our transcriptomic analysis showed different expression patterns of genes involved in ABA biosynthesis and signaling in seedling maize in response to abiotic stresses.

### DEGs Encoding Transcription Factors

Transcription factors regulate almost all aspects of plant growth and development and could orchestrate regulatory networks to improve resistance to abiotic and biotic stresses in plants (Golldack et al., 2014). Major plant TF families such as NAC, AP2/ERF, bZIP, and MYB have been documented as important regulators in plant responses to various abiotic and biotic stresses (Golldack et al., 2011; Shan et al., 2013; Shi et al., 2014; Song et al., 2014; Miao et al., 2015; Shankar et al., 2016; Wang H.Y. et al., 2016). In this study, a total of 43 TF families containing 403 differentially expressed TFs were identified by a paired comparison. Most of the differentially expressed TFs belonged to the ERF, MYB, bZIP, bHLH, WRKY, and NAC TF families (**Figure 4A**). The ERF TF family was the largest class in the seedling maize leaf response to abiotic stresses, with 48 up-regulated and 10 down-regulated DEGs. A previous study identified 184 *AP2/ERF* genes in maize, 38 of which were involved in the response to waterlog stress (Du et al., 2014). Moreover, over-expressing three *DREB1s* belonging to the ERF TF family significantly improved resistance to freezing, drought, and high salinity in *Arabidopsis* (Gilmour et al., 1998; Jaglo-Ottosen et al., 1998; Liu et al., 1998). Numerous MYB TFs play vital roles in cell development, hormone signaling, and cuticular wax biosynthesis in crops (Seo et al., 2009, 2011). We found 18, 22, 14, and 8 differentially expressed *MYB*s in seedling maize leaves in response to salinity, drought, heat, and cold stresses, respectively. Notably, the over-expression of *OsMYB55*, a member of the MYB TF family, improved resistance to heat and drought stresses through activating the expression of resistance-related genes in maize (Casaretto et al., 2016). *ABP9*, which encodes a bZIP TF, could remarkably enhance the resistance to abiotic stresses such as drought, high salt, freezing temperature, and oxidative stresses in *Arabidopsis* (Zhang et al., 2011). In this study, 30 differentially expressed bZIP TFs were identified in response to salinity, drought, heat, and cold stresses, including eight up-regulated DEGs that were found in at least two comparisons of abiotic stress samples (salinity, drought, heat, or cold stress) with the control. These results emphasized that TFs play vital roles in improving resistance to multiple abiotic stresses in maize.

### DEGs Involved in Very-Long-Chain Fatty Acid (VLCFA) and Lipid Signaling

Cuticular wax, a complex mixture of hydrophobic lipids, covers the outermost surfaces of land plants and acts as a protective barrier against abiotic stresses (Bernard and Joubès, 2013; Wang M.L. et al., 2016). Cuticular wax is composed of VLCFAs (C20 to C34) and their derivatives, which are synthesized in the endoplasmic reticulum (ER; Samuels et al., 2008). Cuticular wax biosynthesis begins with the *de novo* C16 or C18 fatty acid synthesis, which is catalyzed by a multi-enzyme complex that includes fatty acid elongases (FAEs), β-ketoacyl-CoA synthase (KCS), β-ketoacyl-CoA reductase (KCR), and FAR, among other components (Lee and Suh, 2013). The expression of wax biosynthesis-related genes, such as *FAR*s, *KCS6*, and *CER1*, can be highly activated by drought stress in plants (Bourdenx et al., 2011; Wang M.L. et al., 2016). In this study, eight *KCS* genes were significantly up-regulated in response to salinity, drought, heat, and cold stresses (**Figure 7**), which suggested that *KCS* is involved in the resistance to abiotic stresses (salinity, drought, heat, or cold stress) in maize. Additionally, two differentially expressed *FAR* genes were identified in response to salinity and heat stresses. As mentioned above, cuticular wax is biosynthesized in the ER (Samuels et al., 2008). In our study, the “Protein processing in endoplasmic reticulum” pathway was significantly enriched in the seedling maize response to drought; moreover, several up-regulated DEGs in the “Protein processing in endoplasmic reticulum” pathway were identified in salinity, heat and cold stress samples (**Supplementary Figure S2**).

Lipid, the interface between the plant cell and the environment, is an essential biomolecule for plant responses to multiple abiotic and biotic stresses (Hou et al., 2016). Steroids (Vriet et al., 2012; Senthil-Kumar et al., 2013), sphingolipids (Lynch, 2012), phospholipids (Darwish et al., 2009), phosphatidylcholines (Tasseva et al., 2004) and other lipids act as signaling molecules that can improve resistance to various stresses in plants (Hou et al., 2016). Six up-regulated DEGs involved in sterol biosynthesis were identified in response to different abiotic stresses; however, the expression levels of these DEGs involved in maize sterol biosynthesis varied based on the abiotic stress applied (**Figure 7**). Most of the DEGs involved in lipid molecule biosynthesis were significantly up-regulated in seedling maize in response to abiotic stresses, while 3 DEGs involved in phospholipid desaturation were down-regulated. The above analysis demonstrated that lipid regulation is a key molecular and biochemical mechanism for resistance to abiotic stresses in seedling maize.

### Common and Unique Molecular Mechanism Responses to Various Abiotic Stresses in Maize

Plants often suffer from different adverse environmental conditions simultaneously (Ahuja et al., 2010), and common mechanisms have evolved to respond to various abiotic stresses. In our dataset, 167 DEGs (57 up-regulated and 110 down-regulated) were identified by comparison between the control and stress sample (**Figure 2**). Many up-regulated DEGs, such as GRMZM2G014392 (*Vp14*), GRMZM6G441368 (F-box protein), and GRMZM2G373522 (Dehydrin), were found in all four stress samples (salinity, drought, heat, and cold), while the functions of most of the down-regulated genes were unknown (**Supplementary Dataset S1**). Ten up-regulated TFs, belonging to five TF families (five ERFs, two NACs, one ARF, one MYB, and one HD-ZIP), and two down-regulated TFs (one bZIP and one MYB-related) were identified in all four stress samples. These results highlighted the important role of TFs in maize resistance to various abiotic stresses. Furthermore, GO enrichment analysis found that 4, 10, and 34 GO terms were over-represented in 4, 3, and 2 stress samples, respectively. These common GO terms were primarily related to plant hormones (ABA and JA), stress response, and fatty acid biosynthetic processes (**Figure 3**). In agricultural production processes, various abiotic stresses typically occur simultaneously (Ahuja et al., 2010); for example, drought stress is often accompanied by salinity stress, and heat stress by drought stress. Pairwise comparisons of identified common DEGs between different stresses demonstrated that the number of DEGs in Salinity vs. Drought (355), Drought vs. Heat (203), and Heat vs. Cold (197) were the top three comparisons, only 66, 116, and 152 common DEGs in Drought vs. Cold, Salinity vs. Heat, and Salinity vs. Cold (**Figure 2B**). The results are consistent with hierarchical cluster analysis. Drought stress, as salt stress, causes osmotic imbalances in the plant tissues (Mahajan and Tuteja, 2005; Huang et al., 2012), plant maybe evolved similar molecular programs to adapt different stresses. This result offers a possible way to improve the multiple-stress tolerance of maize. Although several common DEGs and GO terms were identified in seedling maize in response to different abiotic stresses, most of the obtained DEGs were unique to a particular abiotic stress, which suggested that maize possesses many common and unique molecular mechanisms relating to the resistance to various abiotic stresses.

## Conclusion

In the present study, RNA-seq was applied to detect the global transcriptional changes in seedling maize leaves in response to abiotic stresses. In total, 5,330 DEGs were identified between the control and stress samples. Genes related to hormone metabolism and signaling, TFs, VLCFA biosynthesis and lipid signaling were found to be involved in the resistance to salinity, drought, heat and/or cold stresses in seedling maize. Importantly, 167 DEGs were commonly identified in four seedling maize samples in response to salinity, drought, heat and cold stresses, which suggests that there are many common and unique molecular mechanisms related to the resistance to various abiotic stresses in maize. This study extends the understanding of the molecular mechanisms of maize leaf resistance to abiotic stresses in the seedling stage and will be useful for identifying major candidate genes and molecular markers for improving resistance to abiotic stresses in maize.

## Author Contributions

PL, WC, ZY, and CX conceived the experiment and made the revision of the manuscript. WC, PL, HF, SX, YZ, DL, JW, and YC performed the research and collected data. PL, WC, and SY analyzed the data and wrote the manuscript. All authors reviewed and approved this submission.

## Conflict of Interest Statement

The authors declare that the research was conducted in the absence of any commercial or financial relationships that could be construed as a potential conflict of interest.

## Footnotes

1 ftp://ftp.ensemblgenomes.org/pub/plants/release-31/gff3/zea_mays

2 http://planttfdb.cbi.pku.edu.cn/

3 http://quantprime.mpimp-golm.mpg.de/
