# Supplementary material for: Transcriptomic Profiling of the Maize (Zea mays L.) Leaf Response to Abiotic Stresses at the Seedling Stage
Source: Front Plant Sci. 2017 Mar 1;8:290. doi: 10.3389/fpls.2017.00290 (PMC5331654; doi:10.3389/fpls.2017.00290)
Supplement: Supplementary file 1 [file Data_Sheet_1.DOCX]

Supplementary Material

**Transcriptomic profiling of the maize (*Zea mays* L.) leaf response to abiotic stresses at the seedling stage**

Pengcheng Li^#^, Wei Cao^#^, Huimin Fang, Shuhui Xu, Shuangyi Yin, Yingying Zhang, Dezhou Lin, Jianan Wang, Yufei Chen, Chenwu Xu^*^, Zefeng Yang^*^

Jiangsu Provincial Key Laboratory of Crop Genetics and Physiology/Co-Innovation Center for Modern Production Technology of Grain Crops, Key Laboratory of Plant Functional Genomics of Ministry of Education, Yangzhou University, Yangzhou 225009, China

#: These authors contribute equally to this work.

*Corresponding authors: Zefeng Yang

Email: zfyang@yzu.edu.cn

Chenwu Xu

Email: qtls@yzu.edu.cn

**Supplementary information**

Additional supporting information can be found in the online version of this article:

**Supplementary Table 1-2**

Table S1. Primer information for genes used to validate the FPKM-based expression data using RT-PCR

Table S2. Pearson correlation analysis between samples

**Supplementary Figure 1-2**

Figure S1. Functional classification of the differentially expressed genes based on GO categorization

Figure S2. Pathway assignment of the differentially expressed genes based on KEGG database.

Table S1. Primer information for genes used to validate the FPKM-based expression data using RT-PCR

| Gene Name | Primer sequence(5’-3’) | Product size(bp) | Annealing temperature(℃) |
| --- | --- | --- | --- |
| GRMZM2G014392 | TGTGCATCCAGCTCCAGTTGTTG  AGCAGCAACCAAGATCCCAGAC | 69 | 62.5 |
| GRMZM2G057959 | TCGTTGTTGCGAGAGACAAGCAG  TCCCTTGAATGTTTGGGCAAGGG | 71 | 62.4 |
| GRMZM2G068947 | ACAGAGGAGATACCACACGTCGTC  GCCTGCTTTGATGGCGTTTCTG | 66 | 62.2 |
| GRMZM2G149647 | GGACAACAGTGTCGACGTACAG  ACATCGGATCAACTAACCCGAACG | 134 | 67.5 |
| GRMZM2G117164 | TTCAGAGCCGAGGCAAATGGTG  TGCGATGGAATACGTGCAGAGC | 75 | 62.6 |
| GRMZM2G079440 | GCACTTGCGAGTGGCTTTACTTG  ACCGCTGGAGGTAATATCGACAC | 62 | 59.7 |
| GRMZM2G142802 | TCCTTCCAACCACGAGACTTTGC  AAACAAGGCGGCACACAGATGG | 61 | 60.1 |
| GRMZM2G437100 | TGTGGTACTGTGGTTGCTCTGC  CACGAAACACTGAACAGCACACG | 65 | 60.1 |

Table S2. Pearson correlation analysis between samples

|  | Control-1 | Control-2 | Salinity-1 | Salinity-2 | Heat-1 | Heat-2 | Cold-1 | Cold-2 | Drought-1 | Drought-2 |
| --- | --- | --- | --- | --- | --- | --- | --- | --- | --- | --- |
| Control-1 | 1 |  |  |  |  |  |  |  |  |  |
| Control-2 | 0.95 | 1 |  |  |  |  |  |  |  |  |
| Salinity-1 | 0.90 | 0.91 | 1 |  |  |  |  |  |  |  |
| Salinity-2 | 0.92 | 0.94 | 0.94 | 1 |  |  |  |  |  |  |
| Heat-1 | 0.91 | 0.91 | 0.90 | 0.91 | 1 |  |  |  |  |  |
| Heat-2 | 0.88 | 0.88 | 0.88 | 0.89 | 0.93 | 1 |  |  |  |  |
| Cold-1 | 0.93 | 0.91 | 0.9 | 0.91 | 0.90 | 0.88 | 1 |  |  |  |
| Cold-2 | 0.92 | 0.91 | 0.91 | 0.92 | 0.90 | 0.88 | 0.94 | 1 |  |  |
| Drought-1 | 0.92 | 0.92 | 0.92 | 0.92 | 0.90 | 0.89 | 0.90 | 0.90 | 1 |  |
| Drought-2 | 0.89 | 0.90 | 0.91 | 0.92 | 0.89 | 0.89 | 0.89 | 0.90 | 0.94 | 1 |


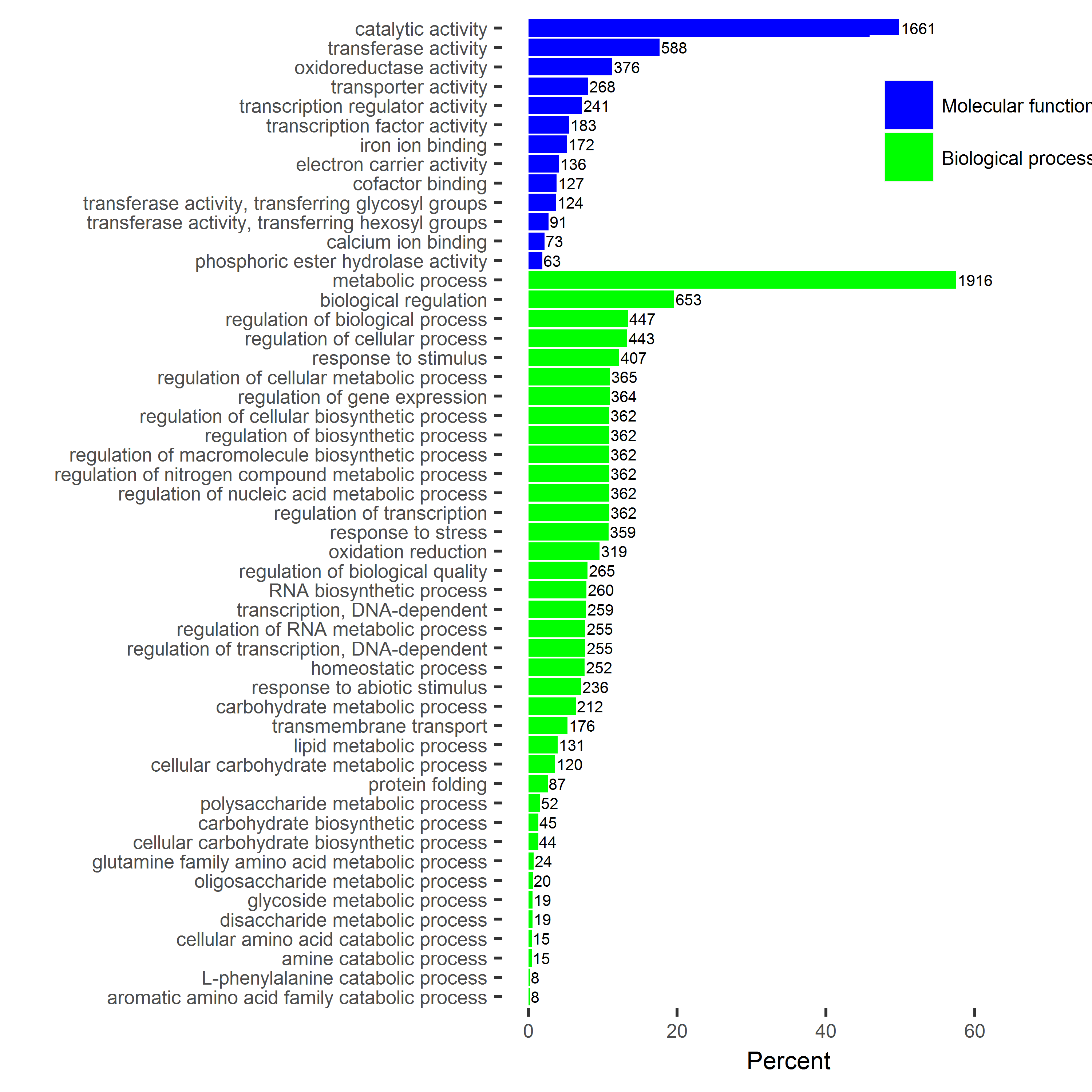


Figure S1. Functional classification of the differentially expressed genes based on GO categorization.


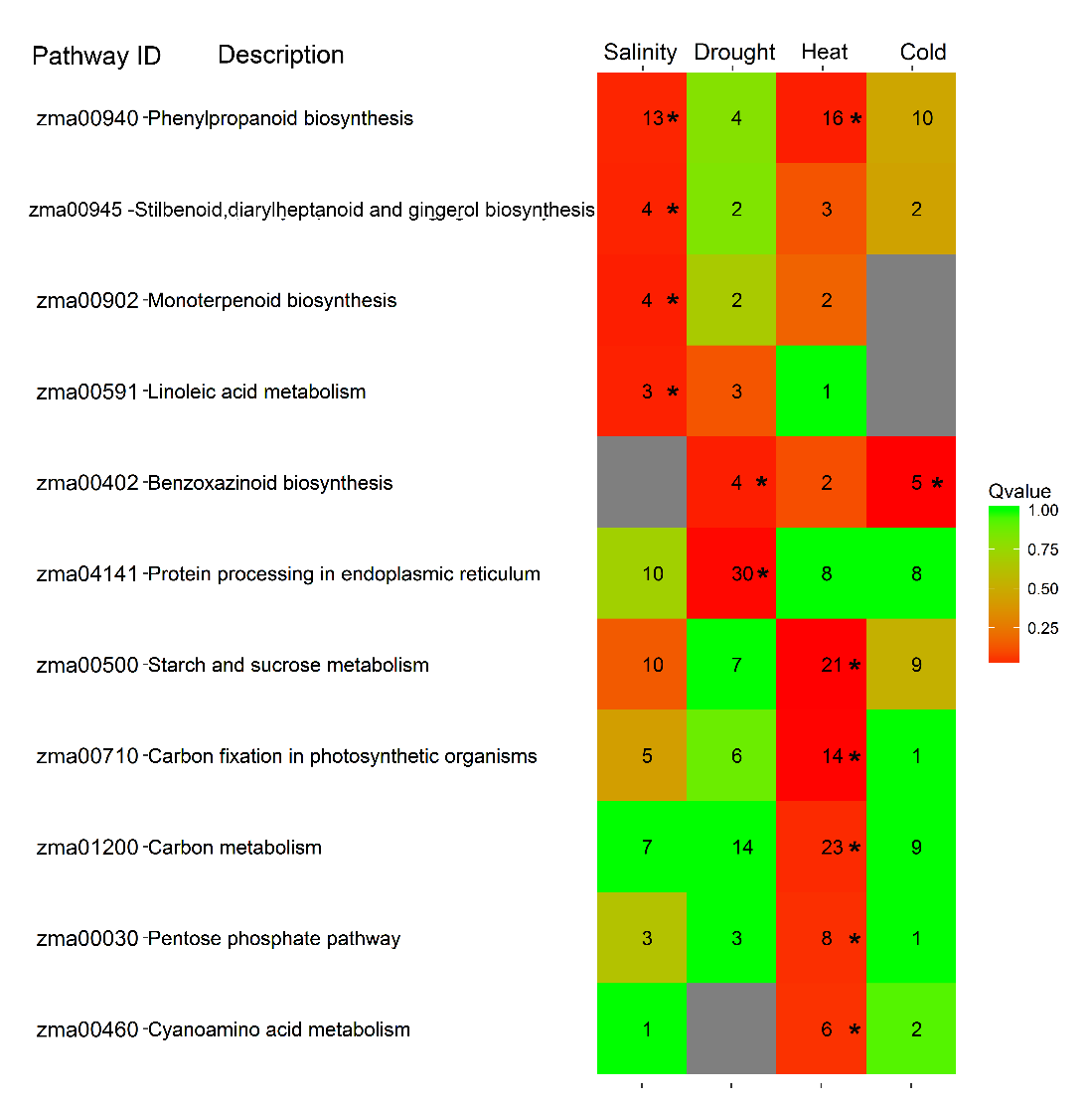


Figure S2. Pathway assignment of the differentially expressed genes based on KEGG database. Different colors in the right columns represent the different significance levels (*q*-values) of the overrepresentation. Asterisks (*) indicated the pathway was significantly overrepresentation.
